# 1-Methyl-3-(2-oxo-2*H*-chromen-3-yl)-1*H*-imidazol-3-ium picrate

**DOI:** 10.1107/S1600536813011690

**Published:** 2013-05-04

**Authors:** Nguyen Van Tuyen, Le Tuan Anh, Alexey A. Festa, Leonid G. Voskressensky, Victor N. Khrustalev

**Affiliations:** a Institute of Chemistry, Vietnam Academy of Science & Technology, 18 Hoang Quoc Viet, Cau Giay, Hanoi, Vietnam; b Department of Chemistry, Vietnam National University, 144 Xuan Thuy, Cau Giay, Hanoi, Vietnam; c Organic Chemistry Department, Russian Peoples Friendship University, Miklukho-Maklai St, 6, Moscow 117198, Russian Federation; d X-Ray Structural Centre, A. N. Nesmeyanov Institute of Organoelement Compounds, Russian Academy of Sciences, Vavilov St 28, B-334, Moscow 119991, Russian Federation

## Abstract

The title salt, C_13_H_11_N_2_O_2_^+^·C_6_H_2_N_3_O_7_^−^, is the unexpected product of a domino reaction of 3-cyano­methyl-1-methyl­imidazolium chloride with salicylic aldehyde in the presence of picric acid. In the cation, the 1*H*-imidazole ring is twisted by 63.2 (1)° from the 2*H*-chromen plane. In the crystal, cations and anions are alternately stacked along the *a* axis through π–π stacking inter­actions between the almost parallel aromatic rings [centroid–centroid distances = 3.458 (2) and 3.678 (2) Å]. The stacks are further linked by C—H⋯O hydrogen bonds into a two-tier layer parallel to (001).

## Related literature

For a recent review on coumarin-based drug patents, see: Kontogiorgis *et al.* (2012 ▶). For analogous domino reactions, see: Voskressensky *et al.* (2012*a* ▶,*b* ▶). For related compounds, see: Yu *et al.* (2006 ▶); Morris *et al.* (2011 ▶).
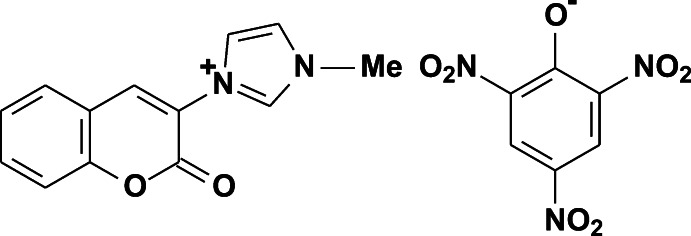


## Experimental

### 

#### Crystal data

C_13_H_11_N_2_O_2_^+^·C_6_H_2_N_3_O_7_^−^
*M* *_r_* = 455.34
Monoclinic, 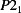

*a* = 6.8142 (12) Å
*b* = 8.1942 (14) Å
*c* = 16.832 (3) Å
β = 100.081 (4)°
*V* = 925.3 (3) Å^3^
*Z* = 2
Mo *K*α radiation
μ = 0.13 mm^−1^
*T* = 100 K
0.30 × 0.21 × 0.03 mm

#### Data collection

Bruker APEXII CCD diffractometer
Absorption correction: multi-scan (*SADABS*; Bruker, 2003 ▶) *T*_min_ = 0.961, *T*_max_ = 0.996
10390 measured reflections
4415 independent reflections
3734 reflections with *I* > 2σ(*I*)
*R*_int_ = 0.040

#### Refinement

*R*[*F* ^2^ > 2σ(*F* ^2^)] = 0.063
*wR*(*F* ^2^) = 0.165
*S* = 1.00
4415 reflections
299 parameters
1 restraint
H-atom parameters constrained
Δρ_max_ = 0.46 e Å^−3^
Δρ_min_ = −0.36 e Å^−3^

### 

Data collection: *APEX2* (Bruker, 2005 ▶); cell refinement: *SAINT* (Bruker, 2001 ▶); data reduction: *SAINT*; program(s) used to solve structure: *SHELXS97* (Sheldrick, 2008 ▶); program(s) used to refine structure: *SHELXL97* (Sheldrick, 2008 ▶); molecular graphics: *SHELXTL* (Sheldrick, 2008 ▶); software used to prepare material for publication: *SHELXTL*.

## Supplementary Material

Crystal structure: contains datablock(s) global, I. DOI: 10.1107/S1600536813011690/is5268sup1.cif

Structure factors: contains datablock(s) I. DOI: 10.1107/S1600536813011690/is5268Isup2.hkl

Supplementary material file. DOI: 10.1107/S1600536813011690/is5268Isup3.cml

Additional supplementary materials:  crystallographic information; 3D view; checkCIF report

## Figures and Tables

**Table 1:** Hydrogen-bond geometry (Å, °)

*D*—H⋯*A*	*D*—H	H⋯*A*	*D*⋯*A*	*D*—H⋯*A*
C5—H5⋯O7^i^	0.95	2.58	3.349 (4)	138
C9—H9⋯O3	0.95	2.33	3.122 (5)	140
C10—H10⋯O9^ii^	0.95	2.51	3.303 (5)	141
C11—H11⋯O3^iii^	0.95	2.42	3.196 (5)	139
C11—H11⋯O5^iii^	0.95	2.51	3.231 (5)	132
C12—H12*A*⋯O2^iv^	0.98	2.58	3.360 (5)	137
C12—H12*B*⋯O2^v^	0.98	2.48	3.448 (5)	171
C12—H12*C*⋯O3^iv^	0.98	2.39	3.269 (4)	148
C12—H12*C*⋯O9^iv^	0.98	2.42	3.160 (5)	132
C17—H17⋯O5^vi^	0.95	2.40	3.345 (5)	172

Symmetry codes: (i) 
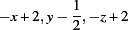
; (ii) 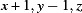; (iii) 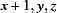; (iv) 
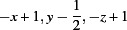
; (v) 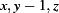; (vi) 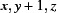.

**Fig. 1.**
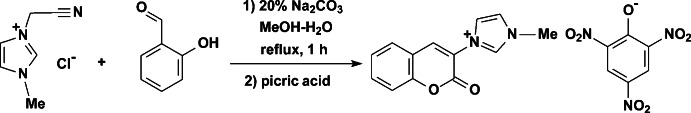
The domino reaction of 3-(cyanomethyl)-1-methylimidazolium chloride with salicylic aldehyde.

**Fig. 2.**
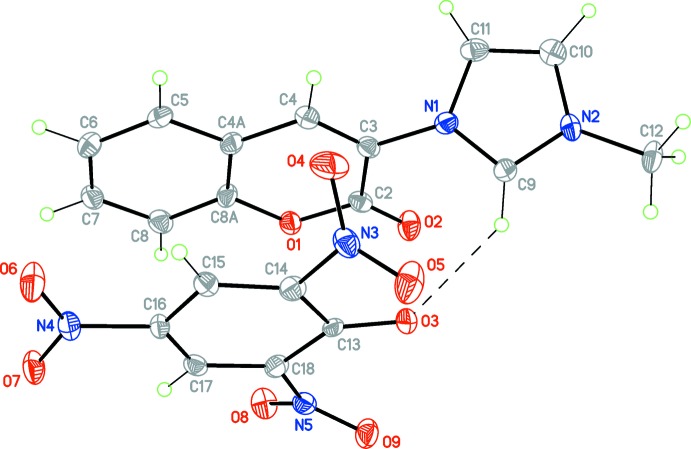
The molecular structure of the title compound. Displacement ellipsoids are shown at the 50% probability level. H atoms are presented as small spheres of arbitrary radius. Dashed line indicates the (N)C(N+)—H···O- hydrogen bond between cation and anion.

**Fig. 3.**
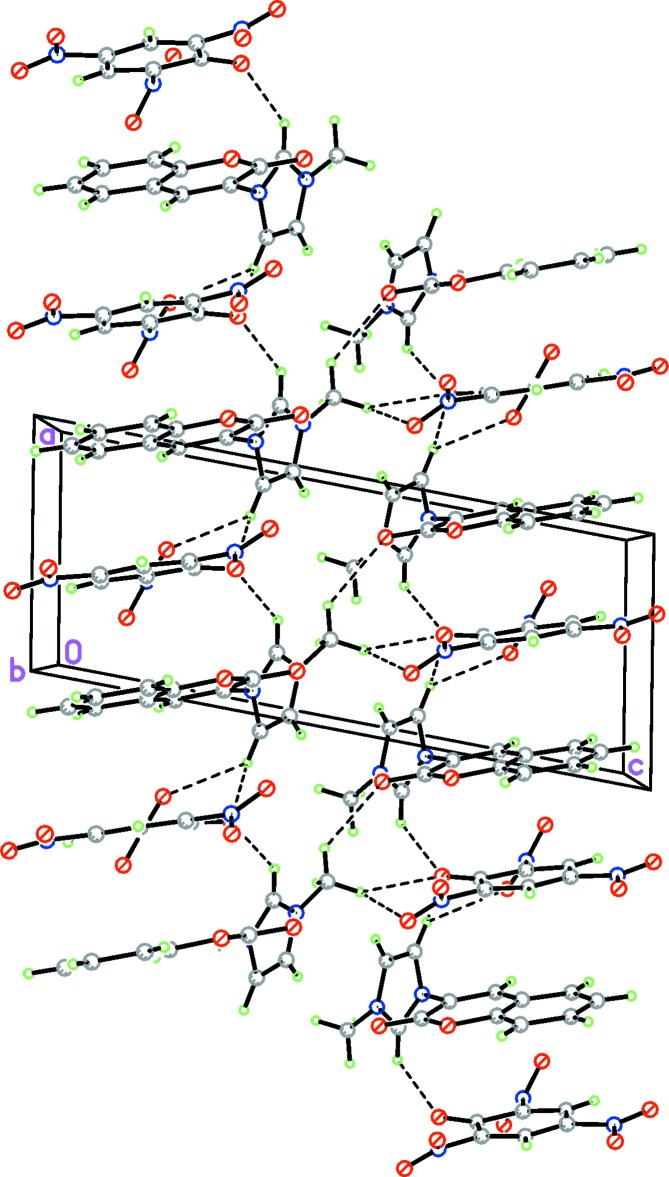
A portion of crystal packing of the title compound demonstrating the stacks along the a axis. Dashed lines indicate the intermolecular C—H···O hydrogen bonds.

**Fig. 4.**
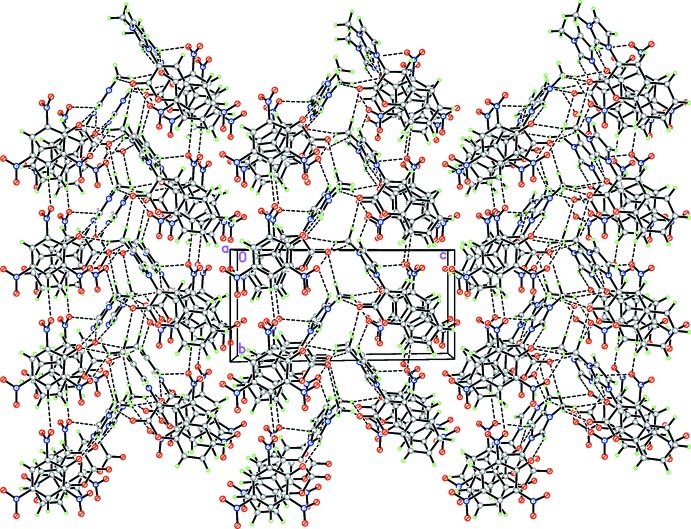
The two-tier layers of the title compound parallel to (001). Dashed lines indicate the intermolecular C—H···O hydrogen bonds.

## supplementary crystallographic information

### Comment

Coumarin derivatives are known to possess a range of different biological activities (Kontogiorgis *et al.*, 2012). The title compound, C_13_H_11_N_2_O_2_^+^.C_6_H_2_N_3_O_7_^-^ (I), is the unexpected product of Knoevenagel condensation of 3-(cyanomethyl)-1-methylimidazolium chloride with salicylic aldehyde followed by the hydrolysis of imino-group and the formation of ammonium salt with picric acid (Fig. 1; Voskressensky *et al.*, 2012*a*,*b*).

The cation and anion of I form a tight ionic pair by the C9—H9···O3 hydrogen bond (Table 1) as well as the π–π stacking interactions between the almost parallel aromatic moieties [the dihedral angle between the mean planes of the 2*H*-chromen (cation) and benzene (anion) fragments is 3.55 (7)°; the shortest C8···C17 distance is 3.280 (5) Å; Fig. 2]. The 1*H*-imidazole ring is twisted at 63.2 (1)° from the 2*H*-chromen plane. In the crystal, the tight ionic pairs form stacks along the *a* axis by the π–π stacking interactions (Fig. 3). The stacks are further bound by the C—H···O hydrogen bonds into two-tier layers parallel to (001) (Fig. 4).

### Experimental

A solid Na_2_CO_3_ (67.0 mg, 0.63 mmol) was added to a stirred solution of 3-(cyanomethyl)-1-methylimidazolium chloride (500 mg, 3.2 mmol) and salicylic aldehyde (350 mg, 2.9 mmol) in a mixture of methanol (4 ml) and water (1 ml) at reflux. The reaction mixture was heated at reflux for 1 h. Then picric acid (870 mg, 3.8 mmol) was added to the solution. The formed precipitate was filtered-off and washed with acetone (3x) to give 630 mg of yellow crystals of I. The yield is 48%. *M*.p. = 459 K (decomp.). ^1^H NMR (DMSO-*d_6_*, 400 MHz): δ = 4.04 (3*H*, s, Me), 7.54 (1*H*, t, *J* = 7.5 Hz, H6'), 7.63 (1*H*, d, *J* = 8.3 Hz, H5'), 7.79–7.85 (1*H*, m, H7'), 7.87–7.92 (1*H*, m, H8'), 7.98–8.01 (1*H*, m, H5), 8.16–8.19 (1*H*, m, H4), 8.61 (2*H*, s, picric acid CH), 8.70 (1*H*, s, H4'), 9.71 (1*H*, bs, H2); ^13^C NMR (DMSO-*d_6_*, 100 MHz): δ = 36.2, 116.4, 117.6, 121.6, 122.5, 123.7, 124.2, 125.1 (2 C), 125.5, 129.4, 133.5, 137.3, 137.5, 141.8, 152.4 (2 C), 156.1, 160.8. Anal. Calcd for C_13_H_11_N_2_O_2_.C_6_H_2_N_3_O_7_: C 50.12, H 2.88, N 15.38; found: C 50.34, H 3.01, N 15.53.

### Refinement

H atoms were placed in calculated positions with C—H = 0.95 Å (CH) and 0.98 Å (CH_3_) and refined in the riding model with fixed isotropic displacement parameters [*U*_iso_(H) = 1.5*U*_eq_(C) for the CH_3_ group and 1.2*U*_eq_(C) for the CH groups].

### Crystal data

**Table:**

C_13_H_11_N_2_O_2_^+^·C_6_H_2_N_3_O_7_^−^	*F*(000) = 468
*M**_r_* = 455.34	*D*_x_ = 1.634 Mg m^−^^3^
Monoclinic, *P*2_1_	Mo *K*α radiation, λ = 0.71073 Å
Hall symbol: P 2yb	Cell parameters from 3018 reflections
*a* = 6.8142 (12) Å	θ = 2.5–30.2°
*b* = 8.1942 (14) Å	µ = 0.13 mm^−^^1^
*c* = 16.832 (3) Å	*T* = 100 K
β = 100.081 (4)°	Plate, yellow
*V* = 925.3 (3) Å^3^	0.30 × 0.21 × 0.03 mm
*Z* = 2	

### Data collection

**Table:**

Bruker APEXII CCD diffractometer	4415 independent reflections
Radiation source: fine-focus sealed tube	3734 reflections with *I* > 2σ(*I*)
Graphite monochromator	*R*_int_ = 0.040
φ and ω scans	θ_max_ = 28.0°, θ_min_ = 2.5°
Absorption correction: multi-scan (*SADABS*; Bruker, 2003)	*h* = −9→9
*T*_min_ = 0.961, *T*_max_ = 0.996	*k* = −10→10
10390 measured reflections	*l* = −22→22

### Refinement

**Table:**

Refinement on *F*^2^	Primary atom site location: structure-invariant direct methods
Least-squares matrix: full	Secondary atom site location: difference Fourier map
*R*[*F*^2^ > 2σ(*F*^2^)] = 0.063	Hydrogen site location: inferred from neighbouring sites
*wR*(*F*^2^) = 0.165	H-atom parameters constrained
*S* = 1.00	*w* = 1/[σ^2^(*F*_o_^2^) + (0.0754*P*)^2^ + 1.86*P*] where *P* = (*F*_o_^2^ + 2*F*_c_^2^)/3
4415 reflections	(Δ/σ)_max_ < 0.001
299 parameters	Δρ_max_ = 0.46 e Å^−^^3^
1 restraint	Δρ_min_ = −0.36 e Å^−^^3^

### Special details

**Table:**

Geometry. All e.s.d.'s (except the e.s.d. in the dihedral angle between two l.s. planes) are estimated using the full covariance matrix. The cell e.s.d.'s are taken into account individually in the estimation of e.s.d.'s in distances, angles and torsion angles; correlations between e.s.d.'s in cell parameters are only used when they are defined by crystal symmetry. An approximate (isotropic) treatment of cell e.s.d.'s is used for estimating e.s.d.'s involving l.s. planes.
Refinement. Refinement of *F*^2^ against ALL reflections. The weighted *R*-factor *wR* and goodness of fit *S* are based on *F*^2^, conventional *R*-factors *R* are based on *F*, with *F* set to zero for negative *F*^2^. The threshold expression of *F*^2^ > σ(*F*^2^) is used only for calculating *R*-factors(gt) *etc*. and is not relevant to the choice of reflections for refinement. *R*-factors based on *F*^2^ are statistically about twice as large as those based on *F*, and *R*- factors based on ALL data will be even larger.

### Fractional atomic coordinates and isotropic or equivalent isotropic displacement parameters (Å^2^)

**Table:**

	*x*	*y*	*z*	*U*_iso_*/*U*_eq_	
O1	0.8777 (4)	0.6678 (3)	0.68430 (15)	0.0183 (5)	
O2	0.8003 (4)	0.5232 (4)	0.57153 (15)	0.0233 (6)	
N1	0.9223 (4)	0.2275 (4)	0.64703 (18)	0.0166 (6)	
N2	0.8021 (5)	0.0278 (4)	0.57071 (17)	0.0166 (6)	
C2	0.8643 (5)	0.5224 (5)	0.6430 (2)	0.0168 (7)	
C3	0.9351 (5)	0.3784 (5)	0.6911 (2)	0.0161 (7)	
C4	1.0105 (5)	0.3861 (5)	0.7702 (2)	0.0171 (7)	
H4	1.0561	0.2899	0.7992	0.021*	
C4A	1.0217 (5)	0.5408 (5)	0.8103 (2)	0.0165 (7)	
C5	1.0926 (5)	0.5595 (5)	0.8937 (2)	0.0178 (7)	
H5	1.1365	0.4666	0.9257	0.021*	
C6	1.0989 (5)	0.7124 (5)	0.9295 (2)	0.0205 (8)	
H6	1.1461	0.7240	0.9857	0.025*	
C7	1.0352 (6)	0.8501 (5)	0.8821 (2)	0.0223 (8)	
H7	1.0420	0.9552	0.9063	0.027*	
C8	0.9623 (6)	0.8333 (5)	0.8001 (2)	0.0208 (8)	
H8	0.9169	0.9257	0.7681	0.025*	
C8A	0.9570 (5)	0.6794 (5)	0.7661 (2)	0.0172 (7)	
C9	0.7529 (5)	0.1610 (5)	0.6077 (2)	0.0163 (7)	
H9	0.6218	0.2018	0.6064	0.020*	
C10	1.0046 (6)	0.0076 (5)	0.5862 (2)	0.0198 (7)	
H10	1.0772	−0.0786	0.5671	0.024*	
C11	1.0826 (5)	0.1338 (5)	0.6341 (2)	0.0200 (7)	
H11	1.2195	0.1536	0.6546	0.024*	
C12	0.6615 (6)	−0.0810 (5)	0.5195 (2)	0.0218 (8)	
H12A	0.5247	−0.0517	0.5245	0.033*	
H12B	0.6878	−0.1942	0.5369	0.033*	
H12C	0.6784	−0.0694	0.4632	0.033*	
O3	0.4485 (4)	0.3812 (3)	0.67659 (15)	0.0185 (5)	
O4	0.7273 (4)	0.1650 (4)	0.85805 (18)	0.0293 (7)	
O5	0.4224 (5)	0.1338 (4)	0.79424 (19)	0.0310 (7)	
O6	0.6970 (5)	0.6752 (4)	1.02204 (16)	0.0283 (7)	
O7	0.5824 (4)	0.8942 (3)	0.95546 (17)	0.0245 (6)	
O8	0.4486 (4)	0.8736 (3)	0.66647 (16)	0.0239 (6)	
O9	0.2690 (4)	0.6684 (4)	0.61602 (16)	0.0257 (6)	
N3	0.5653 (5)	0.2192 (4)	0.82445 (19)	0.0197 (6)	
N4	0.6210 (5)	0.7472 (4)	0.95962 (18)	0.0183 (6)	
N5	0.3876 (5)	0.7341 (4)	0.67024 (19)	0.0185 (6)	
C13	0.4738 (5)	0.4649 (4)	0.7400 (2)	0.0134 (7)	
C14	0.5419 (5)	0.3960 (5)	0.8190 (2)	0.0173 (7)	
C15	0.5962 (5)	0.4837 (4)	0.8896 (2)	0.0155 (7)	
H15	0.6481	0.4310	0.9391	0.019*	
C16	0.5717 (5)	0.6527 (5)	0.8853 (2)	0.0159 (7)	
C17	0.5037 (5)	0.7321 (5)	0.8138 (2)	0.0161 (7)	
H17	0.4908	0.8476	0.8125	0.019*	
C18	0.4542 (5)	0.6419 (5)	0.7437 (2)	0.0170 (7)	

### Atomic displacement parameters (Å^2^)

**Table:**

	*U*^11^	*U*^22^	*U*^33^	*U*^12^	*U*^13^	*U*^23^
O1	0.0207 (13)	0.0180 (13)	0.0157 (12)	0.0025 (11)	0.0020 (9)	−0.0013 (10)
O2	0.0339 (16)	0.0201 (14)	0.0150 (12)	−0.0014 (12)	0.0016 (11)	0.0006 (11)
N1	0.0142 (14)	0.0172 (15)	0.0181 (14)	−0.0001 (12)	0.0019 (11)	−0.0001 (12)
N2	0.0237 (16)	0.0131 (14)	0.0130 (13)	−0.0004 (12)	0.0036 (11)	−0.0013 (11)
C2	0.0155 (17)	0.0147 (16)	0.0212 (17)	0.0007 (14)	0.0062 (13)	0.0011 (14)
C3	0.0155 (16)	0.0133 (16)	0.0195 (17)	0.0007 (13)	0.0035 (13)	−0.0003 (14)
C4	0.0178 (16)	0.0163 (17)	0.0175 (16)	0.0021 (14)	0.0042 (13)	0.0010 (14)
C4A	0.0121 (16)	0.0192 (18)	0.0181 (16)	−0.0026 (14)	0.0024 (13)	−0.0024 (14)
C5	0.0141 (17)	0.0206 (18)	0.0175 (17)	0.0014 (14)	−0.0002 (13)	0.0014 (14)
C6	0.0167 (17)	0.022 (2)	0.0212 (18)	−0.0030 (14)	−0.0002 (13)	−0.0036 (15)
C7	0.0167 (18)	0.022 (2)	0.028 (2)	−0.0007 (15)	0.0040 (15)	−0.0101 (16)
C8	0.0208 (19)	0.0158 (18)	0.0256 (19)	0.0011 (14)	0.0038 (15)	−0.0010 (14)
C8A	0.0176 (17)	0.0215 (18)	0.0117 (15)	−0.0002 (14)	0.0010 (12)	−0.0012 (14)
C9	0.0165 (16)	0.0163 (17)	0.0157 (16)	0.0008 (14)	0.0018 (12)	−0.0001 (13)
C10	0.0222 (18)	0.0217 (19)	0.0169 (16)	0.0009 (15)	0.0072 (13)	0.0022 (14)
C11	0.0163 (17)	0.0250 (19)	0.0192 (18)	0.0025 (15)	0.0047 (13)	0.0031 (14)
C12	0.029 (2)	0.0186 (19)	0.0155 (17)	−0.0051 (15)	−0.0025 (14)	−0.0030 (14)
O3	0.0197 (13)	0.0201 (13)	0.0150 (12)	0.0018 (11)	0.0011 (9)	−0.0049 (11)
O4	0.0266 (15)	0.0243 (15)	0.0361 (16)	0.0091 (12)	0.0032 (12)	0.0054 (13)
O5	0.0455 (18)	0.0140 (14)	0.0279 (15)	−0.0041 (13)	−0.0089 (13)	0.0022 (11)
O6	0.0429 (17)	0.0229 (15)	0.0172 (13)	−0.0014 (13)	0.0000 (12)	−0.0038 (11)
O7	0.0348 (15)	0.0170 (14)	0.0207 (13)	−0.0002 (12)	0.0022 (11)	−0.0069 (11)
O8	0.0346 (16)	0.0162 (13)	0.0212 (13)	−0.0008 (12)	0.0061 (11)	0.0041 (11)
O9	0.0310 (15)	0.0231 (14)	0.0195 (13)	0.0036 (12)	−0.0057 (11)	−0.0036 (11)
N3	0.0268 (17)	0.0144 (15)	0.0179 (15)	0.0034 (13)	0.0041 (12)	0.0003 (12)
N4	0.0204 (15)	0.0182 (16)	0.0172 (15)	−0.0036 (12)	0.0060 (12)	−0.0042 (12)
N5	0.0173 (15)	0.0213 (16)	0.0170 (15)	0.0028 (13)	0.0036 (12)	−0.0008 (12)
C13	0.0079 (16)	0.0186 (18)	0.0135 (15)	−0.0004 (12)	0.0015 (12)	−0.0022 (12)
C14	0.0168 (17)	0.0131 (17)	0.0212 (18)	0.0002 (14)	0.0011 (13)	0.0017 (14)
C15	0.0147 (17)	0.0144 (17)	0.0184 (17)	−0.0013 (13)	0.0051 (13)	−0.0006 (13)
C16	0.0157 (16)	0.0160 (17)	0.0159 (16)	−0.0011 (14)	0.0023 (12)	−0.0057 (14)
C17	0.0129 (16)	0.0159 (17)	0.0205 (17)	−0.0002 (14)	0.0062 (13)	−0.0020 (14)
C18	0.0148 (17)	0.0170 (18)	0.0182 (17)	−0.0003 (14)	0.0002 (13)	0.0003 (14)

### Geometric parameters (Å, º)

**Table:**

O1—C2	1.374 (5)	C10—C11	1.361 (6)
O1—C8A	1.391 (4)	C10—H10	0.9500
O2—C2	1.206 (5)	C11—H11	0.9500
N1—C9	1.342 (5)	C12—H12A	0.9800
N1—C11	1.383 (5)	C12—H12B	0.9800
N1—C3	1.437 (5)	C12—H12C	0.9800
N2—C9	1.328 (5)	O3—C13	1.255 (4)
N2—C10	1.369 (5)	O4—N3	1.232 (4)
N2—C12	1.472 (5)	O5—N3	1.235 (4)
C2—C3	1.464 (5)	O6—N4	1.237 (4)
C3—C4	1.342 (5)	O7—N4	1.232 (4)
C4—C4A	1.433 (5)	O8—N5	1.222 (4)
C4—H4	0.9500	O9—N5	1.232 (4)
C4A—C8A	1.387 (5)	N3—C14	1.458 (5)
C4A—C5	1.410 (5)	N4—C16	1.460 (4)
C5—C6	1.387 (6)	N5—C18	1.452 (5)
C5—H5	0.9500	C13—C14	1.445 (5)
C6—C7	1.405 (6)	C13—C18	1.458 (5)
C6—H6	0.9500	C14—C15	1.383 (5)
C7—C8	1.389 (6)	C15—C16	1.395 (5)
C7—H7	0.9500	C15—H15	0.9500
C8—C8A	1.383 (5)	C16—C17	1.376 (5)
C8—H8	0.9500	C17—C18	1.383 (5)
C9—H9	0.9500	C17—H17	0.9500
			
C2—O1—C8A	122.7 (3)	C11—C10—H10	126.4
C9—N1—C11	109.4 (3)	N2—C10—H10	126.4
C9—N1—C3	125.0 (3)	C10—C11—N1	106.1 (3)
C11—N1—C3	125.5 (3)	C10—C11—H11	126.9
C9—N2—C10	109.8 (3)	N1—C11—H11	126.9
C9—N2—C12	125.5 (3)	N2—C12—H12A	109.5
C10—N2—C12	124.7 (3)	N2—C12—H12B	109.5
O2—C2—O1	118.7 (3)	H12A—C12—H12B	109.5
O2—C2—C3	125.7 (4)	N2—C12—H12C	109.5
O1—C2—C3	115.6 (3)	H12A—C12—H12C	109.5
C4—C3—N1	122.0 (3)	H12B—C12—H12C	109.5
C4—C3—C2	122.9 (4)	O4—N3—O5	124.3 (3)
N1—C3—C2	115.1 (3)	O4—N3—C14	117.8 (3)
C3—C4—C4A	119.3 (4)	O5—N3—C14	117.9 (3)
C3—C4—H4	120.4	O7—N4—O6	124.6 (3)
C4A—C4—H4	120.4	O7—N4—C16	117.1 (3)
C8A—C4A—C5	117.8 (3)	O6—N4—C16	118.3 (3)
C8A—C4A—C4	119.1 (3)	O8—N5—O9	123.7 (3)
C5—C4A—C4	123.1 (4)	O8—N5—C18	118.2 (3)
C6—C5—C4A	120.6 (4)	O9—N5—C18	118.0 (3)
C6—C5—H5	119.7	O3—C13—C14	122.9 (3)
C4A—C5—H5	119.7	O3—C13—C18	125.4 (3)
C5—C6—C7	119.7 (3)	C14—C13—C18	111.5 (3)
C5—C6—H6	120.1	C15—C14—C13	125.6 (3)
C7—C6—H6	120.1	C15—C14—N3	116.9 (3)
C8—C7—C6	120.3 (4)	C13—C14—N3	117.4 (3)
C8—C7—H7	119.8	C14—C15—C16	117.4 (3)
C6—C7—H7	119.8	C14—C15—H15	121.3
C8A—C8—C7	118.7 (4)	C16—C15—H15	121.3
C8A—C8—H8	120.6	C17—C16—C15	122.3 (3)
C7—C8—H8	120.6	C17—C16—N4	119.4 (3)
C8—C8A—C4A	122.8 (3)	C15—C16—N4	118.3 (3)
C8—C8A—O1	116.8 (3)	C16—C17—C18	119.2 (3)
C4A—C8A—O1	120.5 (3)	C16—C17—H17	120.4
N2—C9—N1	107.4 (3)	C18—C17—H17	120.4
N2—C9—H9	126.3	C17—C18—N5	116.2 (3)
N1—C9—H9	126.3	C17—C18—C13	123.9 (3)
C11—C10—N2	107.3 (3)	N5—C18—C13	119.9 (3)
			
C8A—O1—C2—O2	−177.4 (3)	C12—N2—C10—C11	178.7 (3)
C8A—O1—C2—C3	1.3 (5)	N2—C10—C11—N1	0.6 (4)
C9—N1—C3—C4	120.5 (4)	C9—N1—C11—C10	−0.5 (4)
C11—N1—C3—C4	−64.0 (5)	C3—N1—C11—C10	−176.6 (3)
C9—N1—C3—C2	−60.9 (5)	O3—C13—C14—C15	171.5 (4)
C11—N1—C3—C2	114.6 (4)	C18—C13—C14—C15	−4.2 (5)
O2—C2—C3—C4	177.9 (4)	O3—C13—C14—N3	−5.2 (5)
O1—C2—C3—C4	−0.7 (5)	C18—C13—C14—N3	179.1 (3)
O2—C2—C3—N1	−0.7 (5)	O4—N3—C14—C15	−50.5 (5)
O1—C2—C3—N1	−179.4 (3)	O5—N3—C14—C15	130.3 (4)
N1—C3—C4—C4A	179.1 (3)	O4—N3—C14—C13	126.5 (4)
C2—C3—C4—C4A	0.6 (5)	O5—N3—C14—C13	−52.7 (5)
C3—C4—C4A—C8A	−1.0 (5)	C13—C14—C15—C16	4.2 (5)
C3—C4—C4A—C5	177.9 (3)	N3—C14—C15—C16	−179.1 (3)
C8A—C4A—C5—C6	−0.7 (5)	C14—C15—C16—C17	−2.4 (5)
C4—C4A—C5—C6	−179.6 (3)	C14—C15—C16—N4	177.7 (3)
C4A—C5—C6—C7	−0.4 (5)	O7—N4—C16—C17	6.1 (5)
C5—C6—C7—C8	1.3 (6)	O6—N4—C16—C17	−174.2 (3)
C6—C7—C8—C8A	−1.1 (6)	O7—N4—C16—C15	−174.1 (3)
C7—C8—C8A—C4A	−0.1 (6)	O6—N4—C16—C15	5.6 (5)
C7—C8—C8A—O1	178.3 (3)	C15—C16—C17—C18	1.2 (5)
C5—C4A—C8A—C8	1.0 (5)	N4—C16—C17—C18	−179.0 (3)
C4—C4A—C8A—C8	179.9 (4)	C16—C17—C18—N5	−178.7 (3)
C5—C4A—C8A—O1	−177.3 (3)	C16—C17—C18—C13	−1.5 (5)
C4—C4A—C8A—O1	1.6 (5)	O8—N5—C18—C17	27.9 (5)
C2—O1—C8A—C8	179.8 (3)	O9—N5—C18—C17	−150.4 (3)
C2—O1—C8A—C4A	−1.8 (5)	O8—N5—C18—C13	−149.4 (3)
C10—N2—C9—N1	0.1 (4)	O9—N5—C18—C13	32.3 (5)
C12—N2—C9—N1	−179.0 (3)	O3—C13—C18—C17	−172.8 (3)
C11—N1—C9—N2	0.3 (4)	C14—C13—C18—C17	2.8 (5)
C3—N1—C9—N2	176.4 (3)	O3—C13—C18—N5	4.3 (5)
C9—N2—C10—C11	−0.4 (4)	C14—C13—C18—N5	179.9 (3)

### Hydrogen-bond geometry (Å, º)

**Table:**

*D*—H···*A*	*D*—H	H···*A*	*D*···*A*	*D*—H···*A*
C5—H5···O7^i^	0.95	2.58	3.349 (4)	138
C9—H9···O3	0.95	2.33	3.122 (5)	140
C10—H10···O9^ii^	0.95	2.51	3.303 (5)	141
C11—H11···O3^iii^	0.95	2.42	3.196 (5)	139
C11—H11···O5^iii^	0.95	2.51	3.231 (5)	132
C12—H12*A*···O2^iv^	0.98	2.58	3.360 (5)	137
C12—H12*B*···O2^v^	0.98	2.48	3.448 (5)	171
C12—H12*C*···O3^iv^	0.98	2.39	3.269 (4)	148
C12—H12*C*···O9^iv^	0.98	2.42	3.160 (5)	132
C17—H17···O5^vi^	0.95	2.40	3.345 (5)	172

Symmetry codes: (i) −*x*+2, *y*−1/2, −*z*+2; (ii) *x*+1, *y*−1, *z*; (iii) *x*+1, *y*, *z*; (iv) −*x*+1, *y*−1/2, −*z*+1; (v) *x*, *y*−1, *z*; (vi) *x*, *y*+1, *z*.
